# Multi-omic insight into the molecular networks of mitochondrial dysfunction in the pathogenesis of inflammatory bowel disease

**DOI:** 10.1016/j.ebiom.2023.104934

**Published:** 2023-12-16

**Authors:** Jie Chen, Xixian Ruan, Yuhao Sun, Shiyuan Lu, Shixian Hu, Shuai Yuan, Xue Li

**Affiliations:** a Department of Big Data in Health Science, School of Public Health and the Second Affiliated Hospital, Zhejiang University School of Medicine, Hangzhou, Zhejiang, China; b Department of Gastroenterology, The Third Xiangya Hospital, Central South University, Changsha, China; c Department of Gastroenterology, The Second Affiliated Hospital of Zhejiang University School of Medicine, Hangzhou, China; d Department of Gastroenterology, The First Affiliated Hospital, Sun Yat-Sen University, Guangzhou, Guangdong, China; e Institute of Precision Medicine, The First Affiliated Hospital, Sun Yat-Sen University, Guangzhou, Guangdong, China; f Unit of Cardiovascular and Nutritional Epidemiology, Institute of Environmental Medicine, Karolinska Institutet, Stockholm, Sweden

**Keywords:** Inflammatory bowel disease, Mendelian randomization, Mitochondrion, Methylation, Gene expression, Protein

## Abstract

**Background:**

Mitochondrial dysfunction has been linked to the development of inflammatory bowel disease (IBD), but the genetic pathophysiology was not fully elucidated. We employed Mendelian randomization and colocalization analyses to investigate the associations between mitochondrial-related genes and IBD via integrating multi-omics.

**Methods:**

Summary-level data of mitochondrial gene methylation, expression and protein abundance levels were obtained from corresponding methylation, expression and protein quantitative trait loci studies, respectively. We obtained genetic associations with IBD and its two subtypes from the Inflammatory Bowel Disease Genetics Consortium (discovery), the UK Biobank (replication), and the FinnGen study (replication). We performed summary-data-based Mendelian randomization analysis to assess the associations of mitochondrial gene-related molecular features with IBD. Colocalization analysis was further conducted to assess whether the identified signal pairs shared a causal genetic variant.

**Findings:**

After integrating the multi-omics data between mQTL-eQTL and eQTL-pQTL, we identified two mitochondrial genes, i.e., *PARK7* and *ACADM*, with tier 1 evidence for their associations with IBD and ulcerative colitis (UC). *PDK1* and *FISI* genes were associated with UC risk with tier 2 and tier 3 evidence, respectively. The methylation of cg05467918 in *ACADM* was associated with lower expression of *ACADM*, which fits with the positive effect of cg05467918 methylation on UC risk. Consistently, the inverse associations between gene methylation and gene expression were also observed in PARK7 (cg10385390) and PDK1 (cg17679246), which were corroborated with the protective role in UC. At circulating protein level, genetically predicted higher levels of PARK7 (OR 0.36, 95% CI 0.25–0.52) and HINT1 (OR 0.47, 95% CI 0.30–0.74) were inversely associated with IBD risk; genetically predicted higher level of HINT1 was associated with a decreased risk of Crohn's disease (CD) (OR 0.26, 95% CI 0.14–0.49) and a higher level of ACADM (OR 0.67, 95% CI 0.55–0.83), PDK1 (OR 0.63, 95% CI 0.49–0.81), FIS1 (OR 0.63, 95% CI 0.47–0.83) was associated with a decreased risk of UC.

**Interpretation:**

We found that the mitochondrial *PARK7* gene was putatively associated with IBD risk, and mitochondrial *FIS1, PDK1,* and *ACADM* genes were associated with UC risk with evidence from multi-omics levels. This study identified mitochondrial genes in relation to IBD, which may enhance the understanding of the pathogenic mechanisms of IBD development.

**Funding:**

XL is supported by the Natural Science Fund for Distinguished Young Scholars of Zhejiang Province (LR22H260001) and Healthy Zhejiang One Million People Cohort (K-20230085).

**Research in context**

**Evidence before this study**

Although emerging research points towards a pivotal role of mitochondrial dysfunction in the pathogenesis of inflammatory bowel disease (IBD), the comprehensive genetic underpinnings of mitochondrial dysfunction remain to be fully elucidated.

**Added value of this study**

Two mitochondrial genes, including PARK7 and ACADM, were demonstrated with tier 1 evidence of their associations with IBD and UC separately. Moreover, PDK1 and FIS1 displayed evidence of associations with ulcerative colitis risk, backed by tier 2 and tier 3 evidence, respectively.

**Implications of all the available evidence**

The roles of specific mitochondrial genes in IBD pathogenesis were further illuminated, offering promising directions for future research and potential therapeutic targets.

## Introduction

Inflammatory bowel disease (IBD), including Crohn's disease (CD) and ulcerative colitis (UC), causes a considerable health burden.^1^ Mitochondrial function in the gastrointestinal epithelium plays a crucial role in maintaining intestinal health. Growing studies have implicated the involvement of mitochondrial dysfunction in IBD development.^2^ Genome-wide association studies (GWASs) identified *SLC25A28*^3^^,^^4^ and *PARK7*^5^ as IBD susceptibility genes that encode mitochondrial proteins. Besides, experimental studies have shown a significant downregulation of both mitochondrial and nuclear-encoded genes involved in mitochondrial function in patients with UC.^6^ Moreover, before early events during inflammation progression, mitochondrial cristae dissolution or irregularity in CD patients may indicate functional impairment, which leads to altered tight junction function controlling barrier integrity.^7^ Although the crucial role of the mitochondrion in the pathogenesis of IBD has been acknowledged, the specific mitochondrial-related genes and their downstream effects on IBD remain elusive.

Mendelian randomization (MR) analysis uses genetic variants as instrumental variables to enhance inference of the causality between an exposure and an outcome. Compared to observational studies, this method is less susceptible to confounding and reverse causation bias as genetic variants are randomly distributed at conception and cannot be modifiable by the onset of the disease. The increasing availability of large-scale GWAS and molecular quantitative trait loci (QTL) data allows us to explore the causal associations between the regulation of mitochondrial-related genes and IBD at pespectives of methylation, expression, and protein abundance. Here, we utilized MR to investigate the potential associations of mitochondrial gene methylation, expression, and protein abundance with the risk of IBD.

## Methods

**Fig. 1:**
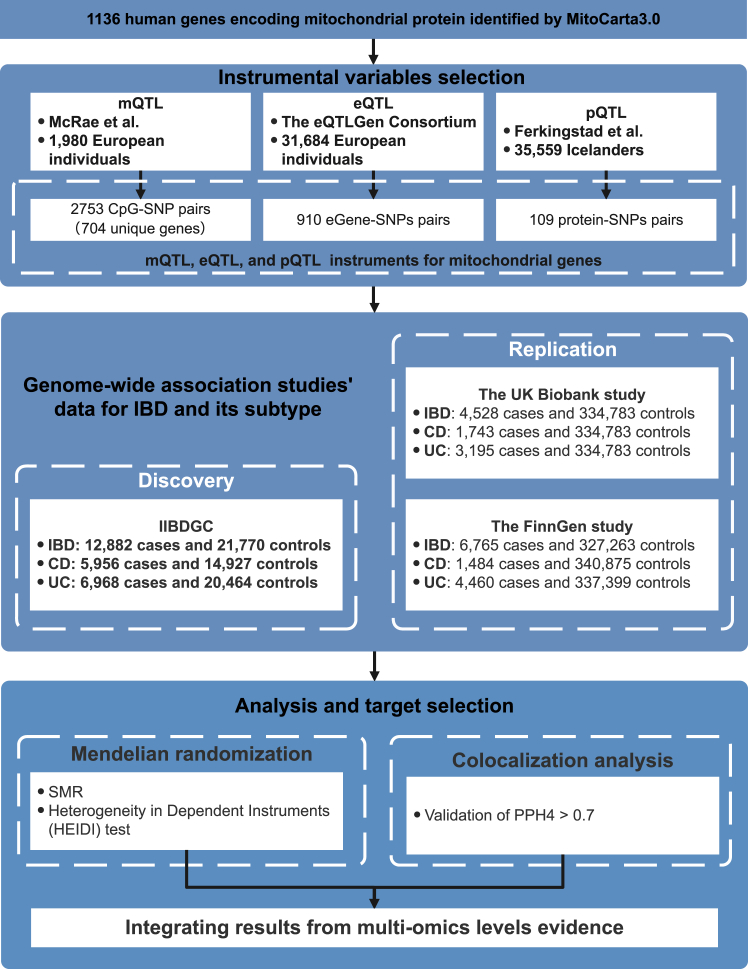
**Study design**. SMR, summary-based Mendelian randomization; QTL, quantitative trait loci; IBD, inflammatory bowel disease; CD, Crohn's disease; UC, ulcerative colitis; SNP, single nucleotide polymorphisms; IIBDGC, the International Inflammatory Bowel Disease Genetics Consortium; PPH4, posterior probability of H4.

Fig. 1 shows the overall design of the study. The current MR analysis was based on publicly available datasets including the International Inflammatory Bowel Disease Genetics Consortium (IIBDGC),^8^ the UK Biobank study,^9^ the FinnGen study^10^ and other large-scale GWASs (Table S1). In this study, instrumental variables for mitochondrial genes were extracted at the methylation, gene expression, and protein abundance levels. Subsequent MR analyses were conducted separately for IBD and its two subtypes at each biological level. To strengthen the causal inference, colocalization analyses were then applied. The IIBDGC dataset served as the primary discovery dataset, and to validate our findings, we utilized the UK Biobank study and the FinnGen study datasets for replication. Specifically, these datasets were employed to investigate gene methylation, gene expression, and protein abundance levels, respectively. Through the integration of results obtained from MR analyses at these three distinct levels, we identified causal candidate genes. There is no overlap in samples between the exposure and outcome populations.

### Data sources of methylation, expression, and protein quantitative trait loci

Integration of multi-omic data enables us to illuminate the underlying molecular networks of mitochondrial dysfunction. QTLs can reveal the associations of single nucleotide polymorphisms (SNPs) with levels of DNA methylation, gene expression, and protein abundance. SNP-CpG associations in blood were obtained from methylation quantitative trait loci (mQTL) data by McRae et al. in 1980 European ancestry individuals.^11^ Individual methylation probes were normalized using a generalized linear model using a logistic link function with corrections for the chip, sex, age, age^2^, sex × age, and sex × age^2^.^11^ The dataset of blood expression quantitative trait loci (eQTL) data was extracted from the eQTLGen consortium which included 31,684 individuals.^12^ Summary statistics of genetic associations with circulating protein levels were extracted from a protein quantitative trait loci (pQTL) study by Ferkingstad et al. comprising 35,559 Icelanders.^13^ Rank-inverse normal transformed levels were adjusted for age, sex, and sample age for each protein tested.^13^

The tissue-specific expression of target genes, demonstrating potential causal effects on IBD, was subsequently assessed by employing tissue-specific expression eQTL data retrieved from the Genotype Tissue Expression (GTEx) web portal (https://gtexportal.org/home/).^14^ The GTEx v8 dataset, comprises 838 donors and 17,382 samples from 52 tissues and two cell lines. In analysis of IBD and CD, we utilized the eQTL data in the sigmoid colon, transverse colon, and small intestine (terminal ileum). For the analysis of UC, we additionally employed the eQTL data in the sigmoid colon and transverse colon.

Mitochondrial-related genes were identified by MitoCarta3.0 which contains an updated inventory of 1136 human mitochondrial genes.^15^ The MitoCarta identified all protein components resident in the mitochondrion based on the Bayesian integration of seven experimental and sequence features. Each mitochondrial gene product was subjected to an independent literature-guided review in the updated MitoCarta3.0, thus providing an inventory of 1136 human mitochondrial genes.^15^ Leveraging the inventory, we separately identified mitochondrial genes in the QTL datasets. After screening for mitochondrial genes, 704 methylation genes, 910 expressed genes, and 109 proteins with available instruments (mQTLs, eQTLs, and pQTLs with P < 5 × 10^−8^) were derived respectively from mQTL, eQTL, and pQTL dataset.

### IBD outcome datasets

Summary-level data for IBD and its two subtypes were obtained from the IIBDGC, the FinnGen study, and UK Biobank studies. All participants in the IIBDGC study were of European ancestry and included 25,042 cases and 34,915 controls for IBD, 12,194 cases and 28,072 controls for CD, and 12,366 cases and 33,609 controls for UC. The genetic associations for CD, UC, and IBD were adjusted for the principal components. Summary statistics of genetic associations with IBD in the UK Biobank were extracted from a GWAS conducted by the Lee Lab.^9^ The IBD diagnoses were defined according to the International Classification of Diseases, 9th Revision (ICD-9) and ICD-10. In total, there were 4528 cases and 334,783 controls for IBD, 1743 cases and 334,783 controls for CD, and 3195 cases and 334,783 controls for UC. Summary-level data of genetic associations with IBD and its subtype were also obtained from the publicly available R8 data release of the FinnGen study. The diagnosis of IBD and its subtype was based on the ICD codes and confirmed by the Social Insurance Institution codes, which totaled 6765 cases and 327,263 controls for IBD, 1484 cases and 340,875 controls for CD and 4460 cases, and 337,399 controls for UC. The discovery stage of the research utilized the IIBDGC dataset, while the replication stage involved the utilization of data from the UK Biobank study and the FinnGen study. There was no sample overlaps among these three datasets.

### Summary-data-based MR analysis

Summary-data-based Mendelian randomization (SMR) was employed to estimate the association of mitochondrial gene methylation, expression, and protein abundance with the risk of IBD and its subtypes.^16^ Based on the top associated cis-QTL, the SMR can reach a much higher statistical power than conventional MR analysis when exposure and outcome are available from two independent samples with large sample sizes.^16^ The top associated cis-QTL were selected by considering a window centered around the corresponding gene (±1000 kb) and passing a P-value threshold of 5.0 × 10^−8^. The SNPs with allele frequency differences larger than the specified threshold (set as 0.2 in the current study) between any pairwise data sets, including the LD reference sample, the QTL summary data, and the outcome summary data, were excluded. Heterogeneity in the dependent instrument (HEIDI) test was applied to distinguish pleiotropy from linkage, where *P*-HEIDI <0.01 were considered likely due to pleiotropy and thus discarded from the analysis. SMR and HEIDI tests were implemented using the SMR software tool (SMR v1.3.1). The P-values were adjusted to control the false discovery rate (FDR) at ɑ = 0.05 using the Benjamini-Hochberg method. Associations with the FDR-corrected P-value <0.05 and P-HEIDI  >0.01 were undertaken for colocalization analysis.

### Colocalization analysis

We conducted colocalization analyses to detect shared causal variants between IBD and identified mitochondrial-related mQTLs, eQTLs, or pQTLs with coloc R package.^17^ In colocalization analysis, five different posterior probabilities are reported, which correspond to the five hypotheses: five exclusive hypotheses: 1) no causal variants for either of the two traits (H0); 2) a causal variant for gene expression only (H1); 3) a causal variant for disease risk only (H2); 4) distinct causal variants for two traits (H3); 5) and the same shared causal variant for both traits (H4). For colocalization of pQTL-GWAS,^18^ eQTL-GWAS^19^ and mQTL-GWAS,^20^ the colocalization region windows were ±1000 kb, ±1000 kb and ±500 kb respectively according to published articles. The prior probabilities that the causal variants is associated with only trait 1 (i.e., mQTL), only trait 2 (i.e., IBD) and both are respectively set at 10^−4^, 10^−4^ and 10^−5^. The posterior probability of H4 (PPH4) >0.70 was considered supporting evidence of colocalization with its cutoff corresponds to a false discovery rate of <5%, which strengthens the evidence for a causal relationship.^21^

### Integrating results from multi-omics level of evidence

To obtain a full picture of associations between regulation of mitochondrial-related genes and IBD at different levels, we integrated results from three different gene regulation tiers. Since proteins are the ultimate expression products of genes and establishing evidence of causation at the protein level is a fundamental requirement, all three tiers of causal candidate genes in our classification are required to have genes causally associated with IBD at the protein level. Based on this principle, we divided the causal candidate genes into three tiers using the following criteria: 1) tier 1 genes were defined to have gene-IBD associations at protein abundance level (FDR-corrected P-value <0.05), PPH4 of colocalization >0.7, and associations with IBD at both methylation and expression levels (FDR-corrected P-value <0.05); 2) tier 2 genes were defined to have gene-IBD association at protein abundance level (FDR-corrected P-value <0.05), PPH4 of colocalization >0.7, and associations with IBD at methylation or expression levels (FDR-corrected P-value <0.05); 3) tier 3 genes were defined to have gene-IBD associations at protein abundance level (FDR-corrected P-value <0.05), PPH4 of colocalization of ≥0.5 and < 0.7, and associations with IBD at both methylation and expression levels (original P-value <0.05). To further explore the potential regulation among gene methylation, expression, and protein abundance, we conducted MR analysis of the causal associations between mitochondrial-related gene methylation and expression, gene expression, and protein abundance. We further performed colocalization analysis for identified associations to rule out the possibility that the association is caused by linkage disequilibrium.

### Ethics

Included studies had been approved by corresponding ethical review committees and all participants signed the consent forms.

### Role of funders

The funders had no role in the study design, data collection, analysis, interpretation, manuscript preparation, or the decision to submit the manuscript for publication.

## Results

### Mitochondrial gene methylation and IBD

**Fig. 2:**
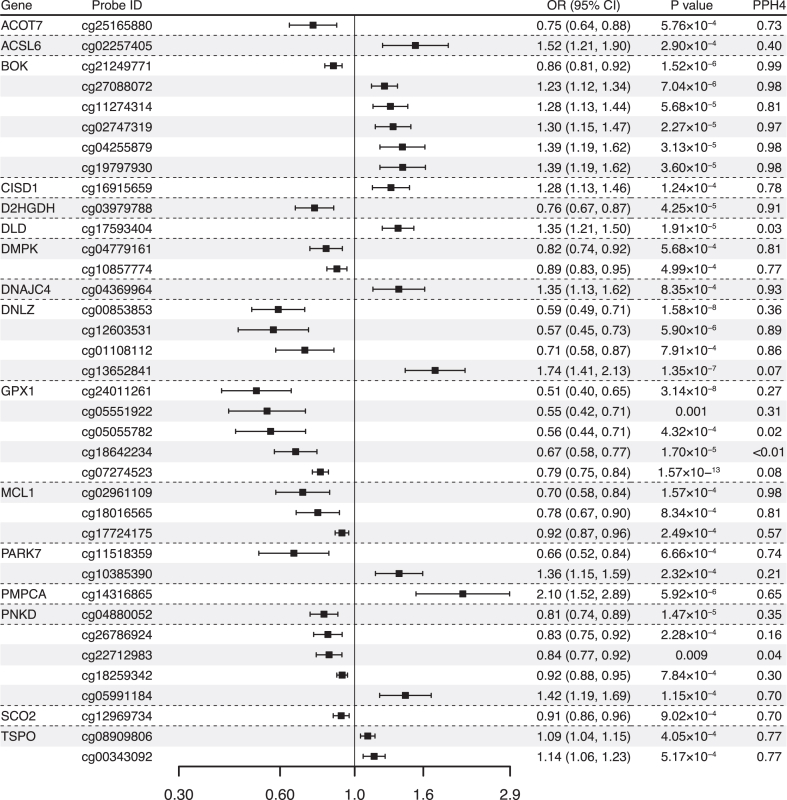
**Associations of genetically predicted mitochondrial gene methylation with inflammatory bowel disease in Mendelian randomization analysis**. OR, odds ratio; CI, confidence interval; PPH4, posterior probability of H4.

**Fig. 3:**
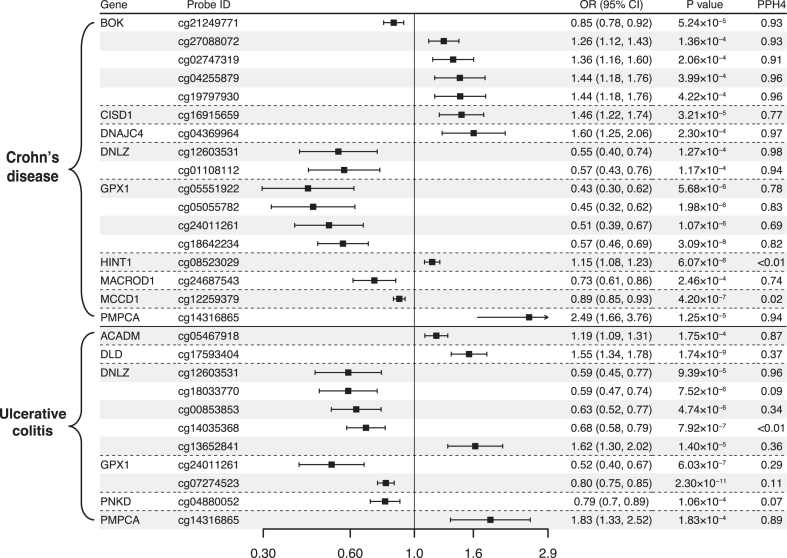
**Associations of genetically predicted mitochondrial gene methylation with Crohn's disease and ulcerative colitis in Mendelian randomization analysis**. OR, odds ratio; CI, confidence interval; PPH4, posterior probability of H4.

Results for causal effects of mitochondrial gene methylation on IBD and its subtype are visualized in Figs. 2 and 3. After the removal of associations with P-HEIDI <0.01, a total of 247 CpG sites near 115 unique genes passed the marginal significance (P < 0.05) (Table S2). After correction for multiple testing, we identified 29 CpG sites near 16 unique genes (Fig. 2). Of the 37 identified signals, 19 near 10 unique genes were found to have strone colocalization evidence support (PPH4 >0.70) including *ACOT* (cg25165880), *BOK* (cg21249771, cg27088072, cg11274314, cg02747319, cg04255879, cg19797930), *D2HGDH* (cg03979788), *DMPK* (cg04779161, cg10857774), *DNAJC4* (cg04369964), *DNLZ* (cg12603531, cg01108112), *MCL1* (cg02961109, cg18016565), *PAKR7* (cg11518359), *SCO2* (cg1296734) and *TSPO* (cg08909806, cg00343092). The direction of effect estimates were not always consistent for different CpG sites located in the same gene. For example, one SD increase in genetically predicted *BOK* methylation at cg21249771 was associated with a decreased risk of IBD (OR 0.86, 95% confidence interval [CI] 0.81–0.92), whereas one SD increase in genetically predicted *BOK* methylation at cg27088072 was associated with a higher risk of IBD (OR 1.23, 95% CI 1.12–1.34). Among these identified CpG sites, the association for cg12603531 near *DNLZ* was replicated in UK Biobank and the associations for cg02961109 and cg18016565 near *MCL1*, cg25165880 near *ACOT,* and cg11518359 near *PARK7* were replicated in FinnGen (Table S3).

Likewise, there were 191 CpG sites associated with CD risk at the P < 0.05 level (Table S4) and 17 CpG sites within 9 unique genes survived after multiple comparison corrections. Colocalization analysis had strong support for cg21249771, cg27088072, cg02747319, cg04255879, cg19797930 near *BOK*, cg04369964 (located in *DNAJC4*), cg01108112 and cg12603531 (located in *DNLZ*), cg18642234 and cg05551922 (located in *GPX1*), cg14316865 (located in *PMPCA*). The association for cg01108112 was replicated in FinnGen (Table S5). There were 218 CpG sites without detecting pleiotropy associated with UC risk (Table S6) and 7 CpG sites with 4 unique genes survived after the FDR correction: *ACADM*, *DNLZ*, *GPX1*, and *PMPCA*. cg05467918 (located in *ACADM*), cg12603531 (located in *DNLZ*), and cg14316865 (located in *PMPCA*) had high support evidence of colocalization. Association for cg12603531 and cg14316865 were replicated in UK Biobank (Table S7). Interestingly, the associations for cg12603531 and cg14316865 were observed for both CD and UC.

### Mitochondrial gene expression and IBD

Results for causal effects of mitochondrial gene expression on IBD and its subtype are presented in Table 1. In total, 67 associations were identified to be associated with IBD at the nominally significant level (P < 0.05) (Table S8). After multiple testing correction and colocalization analysis, genetically predicted higher levels expression of *TUFM* (OR 1.12, 95% CI 1.08–1.17; PPH4 = 0.74), *MTX1* (OR 1.31, 95% CI 1.17–1.47; PPH4 = 0.82), *MRPL20* (OR 1.43, 95% CI 1.21–1.68; PPH4 = 0.83), *CISD1* (OR 1.10, 95% CI 1.05–1.15; PPH4 = 0.72), *BCL2L11* (OR 1.92, 95% CI 1.37–2.69; PPH4 = 0.91) and *PMPCA* (OR 15.12, 95% CI 5.16–44.37; PPH4 = 0.72) were positively associated with IBD risk. Conversely, genetically predicted higher levels expression of *PARK7* (OR 0.71, 95% CI 0.63–0.79) were inversely associated with IBD risk (Table 1). Association for *BCL2L11* was replicated in FinnGen and UK Biobank; Associations for *TUFM MRPL20, CISD1* and *PMPCA* were replicated in FinnGen (Table S9).

**Table 1:** Associations of genetically predicted mitochondrial gene expression with inflammatory bowel disease and its subtype in Mendelian randomization analysis.

Disease	Gene	OR (95% CI)	P-value	PPH4
Inflammatory bowel disease	GPX1	0.23 (0.16, 0.34)	2.05 × 10^−13^	0.10
	FDPS	0.40 (0.26, 0.63)	7.23 × 10^−5^	0.37
	MRPL23	0.58 (0.45, 0.75)	2.32 × 10^−5^	0.01
	PARK7	0.71 (0.63, 0.79)	3.31 × 10^−9^	0.91
	CISD1	1.10 (1.05, 1.15)	5.22 × 10^−5^	0.72
	TUFM	1.12 (1.08, 1.17)	2.44 × 10^−6^	0.74
	MTX1	1.31 (1.17, 1.47)	5.96 × 10^−6^	0.82
	MRPL20	1.43 (1.21, 1.68)	1.76 × 10^−5^	0.83
	BCL2L11	1.92 (1.37, 2.69)	1.51 × 10^−4^	0.91
	PMPCA	15.12 (5.16, 44.37)	1.72 × 10^−4^	0.72
Crohn's disease	FDPS	0.22 (0.11, 0.41)	2.39 × 10^−6^	0.76
	GPX1	0.23 (0.14, 0.38)	2.67 × 10^−9^	0.70
	BAD	0.88 (0.82, 0.95)	3.97 × 10^−4^	0.66
	DAP3	0.55 (0.43, 0.70)	1.98 × 10^−6^	0.50
	MARS2	0.60 (0.48, 0.75)	6.40 × 10^−6^	0.69
	MFN1	0.75 (0.65, 0.87)	1.35 × 10^−4^	0.56
	ATP5MD	0.88 (0.82, 0.95)	5.62 × 10^−4^	0.48
	CISD1	1.17 (1.10, 1.24)	1.33 × 10^−6^	0.75
	TUFM	1.18 (1.22, 1.25)	2.40 × 10^−9^	0.74
	HEBP1	1.29 (1.14, 1.46)	6.10 × 10^−5^	0.57
	MTX1	1.37 (1.17, 1.61)	8.00 × 10^−5^	0.01
	PMPCA	28.26 (7.13, 111.90)	1.95 × 10^−6^	0.77
Ulcerative colitis	GPX1	0.24 (0.15, 0.38)	6.60 × 10^−10^	0.64
	MRPL23	0.48 (0.35, 0.66)	4.75 × 10^−6^	0.78
	NDUFAF7	0.64 (0.50, 0.83)	6.55 × 10^−4^	0.93
	GLDC	0.71 (0.60, 0.85)	1.87 × 10^−4^	0.89
	PDK1	0.73 (0.62, 0.87)	2.72 × 10^−4^	0.85
	ME2	0.75 (0.63, 0.89)	7.99 × 10^−4^	0.11
	ACADM	0.78 (0.69, 0.89)	1.22 × 10^−4^	0.61
	ABHD11	0.83 (0.75, 0.92)	3.59 × 10^−4^	0.93
	SLC25A11	1.40 (1.15, 1.71)	9.25 × 10^−4^	<0.01
	MRPL20	1.49 (1.22, 1.82)	8.37 × 10^−5^	0.70
	BCL2L11	2.22 (1.45, 3.39)	2.35 × 10^−4^	0.75
	REXO2	3.22 (1.84, 5.63)	4.09 × 10^−5^	0.24
	PMPCA	8.73 (2.97, 25.64)	8.06 × 10^−5^	0.64

With adjustment for multiple testing and checking for colocalization evidence (PPH4 >0.7), genetically predicted one SD increased in *PMPCA* (OR 28.26, 95% CI 7.13–111.90), *TUFM* (OR 1.18, 95% CI 1.22–1.25) and *CISD1* (OR 1.17, 95% CI 1.10–1.24) expression were associated with increased CD risk; however, genetically predicted one SD increased in *FDPS* (OR 0.22, 95% CI 0.11–0.41) and *GPX1* (OR 0.23, 95% CI 0.14–0.38) were associated with decreased CD risk (Table 1 and Table S10). *PMPCA*, *TUFM* and *GPX1* was replicated in the FinnGen study; *FDPS* was replicated in both datasets (Table S11). After the FDR correction and filtering for colocalization evidence, genetically predicted one SD increase in *MRPL20* (OR 1.49, 95% CI 1.22–1.82), *BCL2L11* (OR 2.22, 95% CI 1.45–3.39) and *PMPCA* (OR 8.73, 95% CI 2.97–25.64) expression were associated with increased UC risk, whereas, genetically predicted one SD increased in *MRPL23* (OR 0.48, 95% CI 0.35–0.66), *NDUFAF7* (OR 0.64, 95% CI 0.50–0.83), *GLDC* (OR 0.71, 95% CI 0.60–0.85), *ABHD11* (OR 0.83, 95% CI 0.75–0.92) were associated with decreased UC risk (Tables 1 and Table S12). *MRPL23* and *ABHD11* were replicated in the UK Biobank study and the FinnGen study; *MRPL20*, *MRPL23*, *BCL2L11* and *PMPCA* were replicated in the FinnGen study (Table S13).

### Mitochondrial protein and IBD

**Fig. 4:**
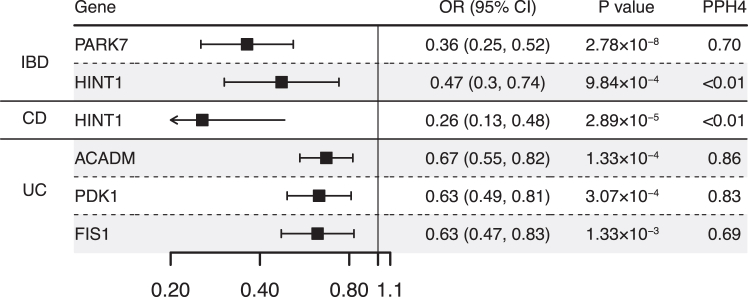
**Associations of genetically predicted mitochondrial gene encoded protein with inflammatory bowel disease and its subtype in Mendelian randomization analysis**. OR, odds ratio; CI, confidence interval; PPH4, posterior probability of H4.

There were 11, 9, and 12 mitochondrial proteins separately associated with IBD, CD, and UC risk at P < 0.05 level (Table S14–S16). After adjustment for multiple testing, genetically predicted higher levels of PARK7 (OR 0.36, 95% CI 0.25–0.52) and HINT1 (OR 0.47, 95% CI 0.30–0.74) were inversely associated with IBD risk (Fig. 4). Genetically predicted higher level of HINT1 was associated with a decreased risk of CD (OR 0.26, 95% CI 0.14–0.49) and a higher level of ACADM (OR 0.67, 95% CI 0.55–0.83), PDK1 (OR 0.63, 95% CI 0.49–0.81), FIS1 (OR 0.63, 95% CI 0.47–0.83) was associated with a decreased risk of UC (Fig. 4). The colocalization evidence was observed between PARK7 and IBD (PPH4 = 0.70). ACADM (PPH4 = 0.86), and PDK1 (PPH4 = 0.83) had high support evidence of colocalization in UC.

### Integrating evidence from multi-omics levels

**Fig. 5:**
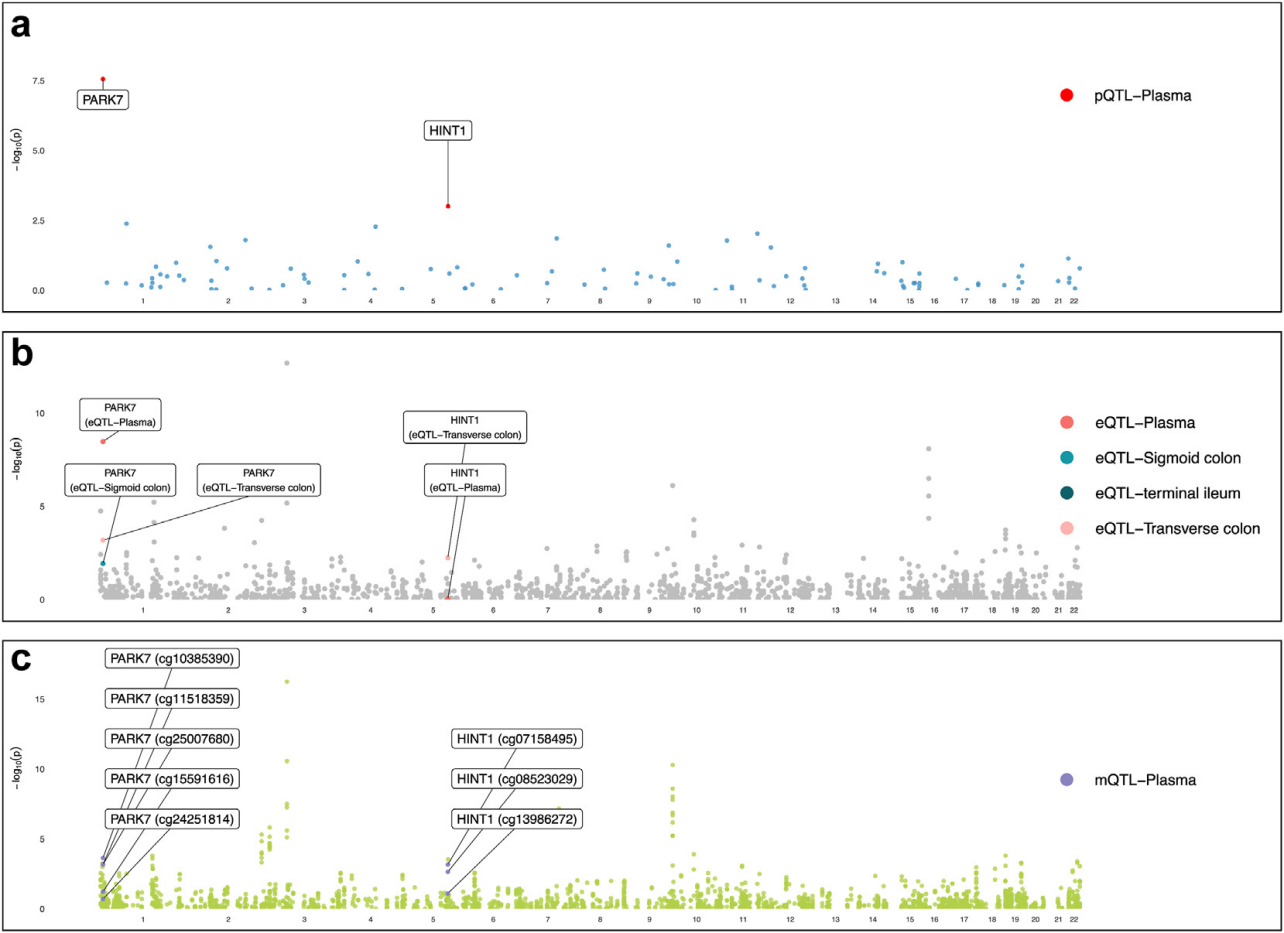
**Manhattan plot for associations between mitochondrial-related gene molecular features and inflammatory bowel disease**. Manhattan plot for mitochondrial-related gene methylation (**a**), expression (**b**) and protein abundance (**c**). Genes with significant signals in protein abundance levels were labeled.

**Fig. 6:**
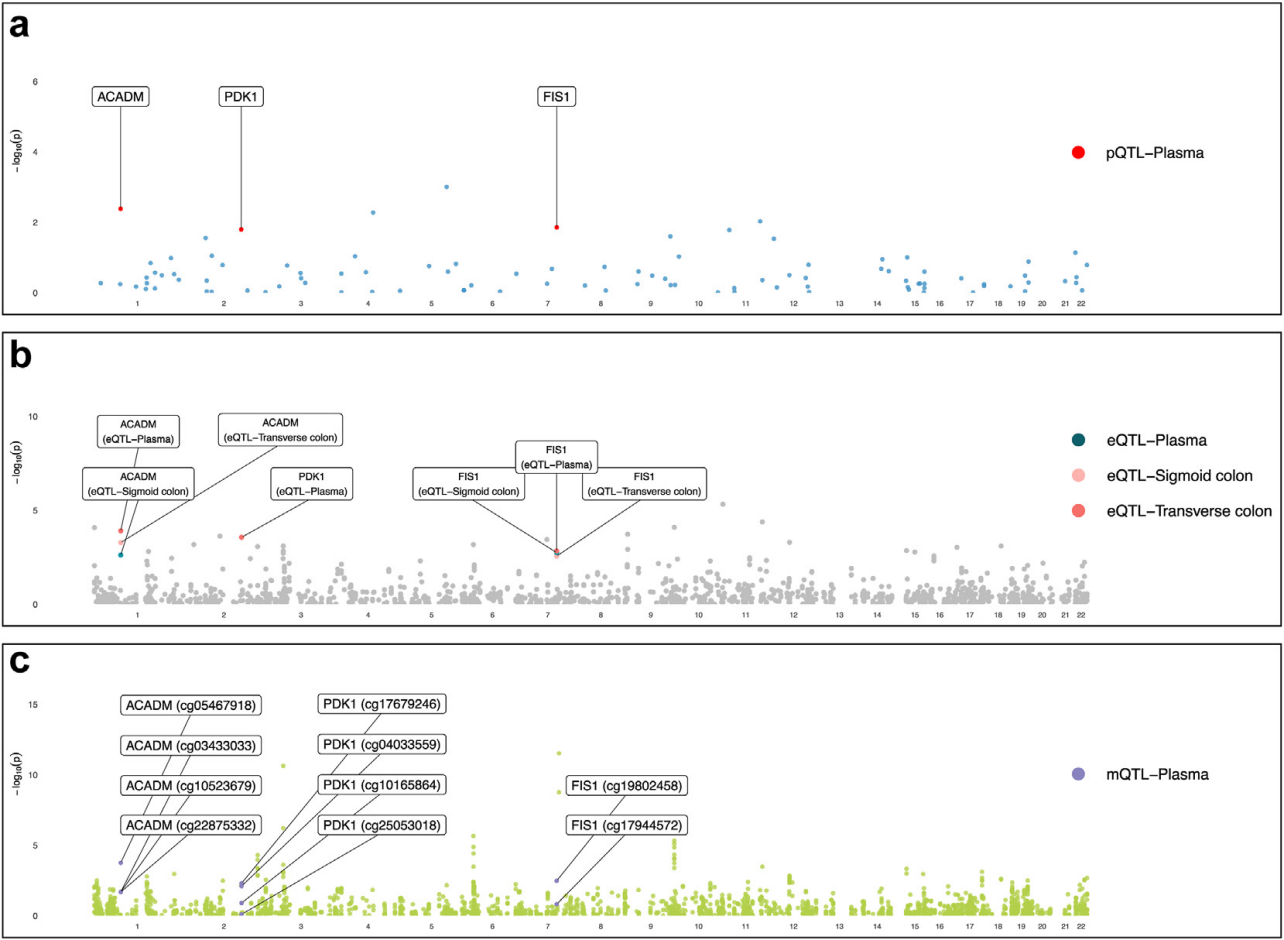
**Manhattan plot for associations between mitochondrial-related gene molecular features and ulcerative colitis**. Manhattan plot for mitochondrial-related gene methylation (**a**), expression (**b**) and protein abundance (**c**). Genes with significant signals in protein abundance levels were labeled.

After the integration of evidence from multi-omics, we identified two genes with tier 1 multi-omics’ evidence, including *PARK7* and *ACADM,* for their associations with IBD and UC. *PDK1* was identified as a tier 2 gene for UC and FISI was identified as a tier 3 gene for UC (Table 2, Figs. 5 and 6). In the replication of the target gene-IBD relationship in the UK Biobank and FinnGen study, it is noteworthy that, while not all associations achieved statistical significance, the majority of associations maintained a consistent direction, aligning with our observations from the discovery stage (Table S17). Being consistent, the expression of 4 identified genes was positively associated with corresponding protein levels in MR analysis (Table S18). The methylation of cg05467918 in *ACADM* was associated with lower expression of *ACADM*, which fitted with the positive effect of cg05467918 methylation on UC (Fig. 2). Similarly, the inverse associations between gene methylation and gene expression were also observed in *PARK7* (cg10385390) and *PDK1* (cg17679246), which were corroborated with the protective role in IBD and UC. The colocalization analysis found strong evidence (PPH4 >0.70) between mQTL-eQTL and eQTL-pQTL for these genes except the colocalization between cg04033559 near *PDK1* and expression of *PDK1* (Table S18).

**Table 2:** Tier of genetically predicted methylation, expression, and protein of candidate gene with IBD and its subtype in Mendelian randomization analysis.

Outcome	Gene	Tier	mQTL			eQTL		pQTL	
			Probe	OR (95% CI)	P-value	OR (95% CI)	P-value	OR (95% CI)	P-value
IBD	PARK7	Tier 1	cg10385390	1.36 (1.15, 1.59)	2.32E-04^a^	0.71 (0.63, 0.79)	3.31E-09^a^	0.36 (0.25, 0.52)	2.78E-08^a^
	PARK7		cg11518359	0.66 (0.52, 0.84)	6.66E-04^a^				
UC	ACADM	Tier 1	cg05467918	1.05 (1.05, 1.05)	1.75E-04^a^	0.78 (0.69, 0.89)	1.22E-04^a^	0.67 (0.55, 0.82)	1.33E-04^a^
	PDK1	Tier 2	cg17679246	1.12 (1.04, 1.22)	4.99E-03	0.73 (0.62, 0.87)	2.72E-04^a^	0.63 (0.49, 0.81)	3.07E-04^a^
	PDK1		cg04033559	0.96 (0.92, 0.99)	7.88E-03				
	FIS1	Tier 3	cg19802458	0.74 (0.61, 0.91)	3.36E-03	0.86 (0.78, 0.94)	1.46E-03	0.63 (0.47, 0.83)	1.13E-03^a^

IBD, inflammatory bowel disease; UC, ulcerative colitis.

a Significant after multiple testing.

### Tissue-specific validation

We further explored causal associations of the expression of identified genes with IBD or UC in bowel tissues. Due to no instruments for PDK1 and FIS1 in the outcome data, the analysis did not include these two genes. Genetically predicted expression levels of *PARK7* were inversely associated with IBD risk in sigmoid colon (OR 0.69, 95% CI 0.52–0.92; P = 0.014) and transverse colon tissue (OR 0.67, 95% CI 0.53–0.84; P = 6.56 × 10^−4^) (Table S19). Instrument of expression of PARK7 in small intestine was missing in the IBD dataset. Genetically predicted expression levels of ACADM were associated with a decreased IBD risk in sigmoid colon (OR 0.78, 95% CI 0.67–0.91; P = 0.002) and transverse colon tissue (OR 0.72, 95% CI 0.60–0.87; P = 5.15 × 10^−4^) (Table S19).

## Discussion

In this study, we performed MR and colocalization analyses to explore the associations of genetically predicted mitochondrial gene methylation, expression, and protein abundance levels with IBD and its subtype. We found that the mitochondrial *PARK7* gene was putatively associated with IBD risk, and mitochondrial *FIS1, PDK1,* and *ACADM* genes were associated with UC with multi-omic evidence.

In the analysis of mQTL, eQTL, and pQTL, *PARK7* was found to be associated with IBD. *PARK7* encodes Parkinson's disease 7 (PARK7), which has a reported protective role in the neurodegenerative diseases^22^ and a well-defined role in mitochondrial homeostasis and mitophagy.^23^ Previous GWASs had identified PARK7 as one of the susceptibility loci associated with IBD.^5^^,^^24^^,^^25^ PARK7 protein has been found to be down-regulated in patients with CD and UC. Recent studies have shown that PARK7 plays an important role in maintaining the gut microbiome and regulating intestinal inflammation via protecting the integrity of the mucosal epithelial barrier.^26^^,^^27^ In detail, deficiency of PARK7 may increase p53 levels and finally promotes intestinal epithelial cell apoptosis. Besides, a previous MR study found that methylation of the cg10385390 increased the risk for IBD by reducing PARK7 expression though lacking validation in protein abundance levels. Results from the current MR study extended and refined this finding and additionally identified that methylation of cg11518359 was positively associated with IBD risk via increasing the expression of PARK7.

The *ACADM* gene encodes the acyl-coenzyme A dehydrogenase (ACADM), which is important for degrading medium-chain fatty acids. In our study, genetically predicted levels of ACADM methylation, expression, and the corresponding protein were associated with UC. Our previous proteome-wide MR analysis of IBD has uncovered that UC had high support for colocalization with ACADM.^28^ However, evidence on the association between ACADM and UC from observational epidemiological and experimental studies was scarce. Based on the current findings, methylation of cg05467918 may decrease the expression of ACADM and thus increase the risks of UC.

Pyruvate dehydrogenase kinase 1 (PDK1) was encoded by *PDK1*, which plays a key role in regulating glucose and fatty acid metabolism.^29^ The protein has also been involved in cellular responses to hypoxia, promoting cell proliferation and protecting against apoptosis induced by hypoxia and oxidative stress.^30^ Experimental studies found that AKT was activated by PDK1 by phosphorylation at T308 residue and increased phosphorylation AKT levels were associated with UC risk.^31^ Our MR analysis provided evidence that the downregulation of cg17679246 methylation was associated with a decreased UC risk via increasing PDK1 expression levels. The *FIS1* gene encodes the mitochondrial fission 1 protein (FIS1), which plays a crucial role in mediating mitochondrial fission. This process is vital for generating a sufficient number of mitochondria to support the growth and function of cells. Interestingly, experimental studies suggest that excessive mitochondria fragmentation may promote the inflammation of intestinal epithelial cells and inhibition of FIS1 protein can prevent colitis by maintaining enterocyte and macrophage mitochondrial networks.^32^^,^^33^ However, inherent biases in observational data could not be avoided. The low mitochondrial division capacity may leads to an insufficient supply of nutrients and energy, resulting in cellular hypoxia and metabolic stress. Findings from the current MR investigation suggested that the methylation of cg19802458 might increase the expression of FIS1 and thus decrease the UC risks. Further studies are needed to better elucidate the role of FIS1 in IBD.

An advantage of our investigation is that we utilized MR and colocalization, two methods that together utilize genetic variants to estimate the causal effects of mitochondrial gene methylation expression and protein abundance. Moreover, we integrated results from multi-omics levels evidence which strengthened the causal relationship between mitochondrial-related genes and IBD risks. The MR design complementarily minimized bias from confounding and reverse causality, thereby improving causal inference, while the colocalization approaches have proven to be a powerful tool in eliminating potential bias caused by linkage disequilibrium. Besides, GWASs with large sample sizes helped increase the statistical power of our study. Furthermore, the consistency of our results across multiple datasets provided additional support for our findings. It is also important to acknowledge the limitations of our study. Due to the limited number of mitochondrial-related proteins in the pQTL dataset, the current study did not fully explore the causal relationship between mitochondrial proteins and the risk of IBD. A further constraint of the study is the interpretation of the posterior probabilities (PPH4) in colocalization should be approached with caution, as a low PPH4 may not necessarily indicate a lack of evidence for colocalization when PPH3 is also low due to insufficient statistical power.^17^

The current MR study explored the potential causal relationships of mitochondrial-related gene methylation, expression, and protein abundance with IBD and demonstrated the importance of several mitochondrial-related genes and their regulation in the pathogenesis of IBD. These findings deepen the pathological understanding of IBD and potentially reveal pharmacological targets.

## Contributors

Jie Chen (Conceptualization: Equal; Methodology: Equal; Formal analysis: Equal; Data curation: Equal; and Writing - review & editing: Equal).

Xixian Ruan (Conceptualization: Equal; Methodology: Equal; Formal analysis: Equal; Data curation: Equal; and Writing—original draft: Equal).

Yuhao Sun (Conceptualization: Equal; Methodology: Equal; Formal analysis: Equal; Data curation: Equal; and Writing—original draft: Equal).

Shiyuan Lu (Conceptualization: Supporting; Writing–review & editing: Supporting).

Shixian Hu (Conceptualization: Equal; Methodology: Equal; Writing–review & editing: Equal).

Shuai Yuan (Conceptualization: Leading; Data curation: Equal; Writing–review & editing: Leading).

Xue Li (Conceptualization: Equal; Data curation: Equal; Funding acquisition: Leading; and Writing–review & editing: Leading).

XL and SY verified the underlying data. All authors read and approved the final manuscript.

## Data sharing statement

Data can be obtained upon a reasonable request to corresponding authors.

## Declaration of interests

The authors declare that they have no competing interests.
